# Disrupted mitochondrial homeostasis coupled with mitotic arrest generates antineoplastic oxidative stress

**DOI:** 10.1038/s41388-021-02105-9

**Published:** 2021-11-12

**Authors:** Xiaohe Hao, Wenqing Bu, Guosheng Lv, Limei Xu, Dong Hou, Jing Wang, Xiaojie Liu, Tingting Yang, Xiyu Zhang, Qiao Liu, Yaoqin Gong, Changshun Shao

**Affiliations:** 1 MOE Key Laboratory of Experimental Teratology, Department of Genetics, Shandong University School of Basic Medical Sciences, Jinan, Shandong 250012 China; 2 State Key Laboratory of Radiation Medicine and Protection, Institutes for Translational Medicine, Soochow University, Suzhou, Jiangsu 215123 China

**Keywords:** Cancer metabolism, Checkpoint signalling

## Abstract

Reactive oxygen species (ROS) serve as critical signals in various cellular processes. Excessive ROS cause cell death or senescence and mediates the therapeutic effect of many cancer drugs. Recent studies showed that ROS increasingly accumulate during G2/M arrest, the underlying mechanism, however, has not been fully elucidated. Here, we show that in cancer cells treated with anticancer agent TH287 or paclitaxel that causes M arrest, mitochondria accumulate robustly and produce excessive mitochondrial superoxide, which causes oxidative DNA damage and undermines cell survival and proliferation. While mitochondrial mass is greatly increased in cells arrested at M phase, the mitochondrial function is compromised, as reflected by reduced mitochondrial membrane potential, increased SUMOylation and acetylation of mitochondrial proteins, as well as an increased metabolic reliance on glycolysis. CHK1 functional disruption decelerates cell cycle, spares the M arrest and attenuates mitochondrial oxidative stress. Induction of mitophagy and blockade of mitochondrial biogenesis, measures that reduce mitochondrial accumulation, also decelerate cell cycle and abrogate M arrest-coupled mitochondrial oxidative stress. These results suggest that cell cycle progression and mitochondrial homeostasis are interdependent and coordinated, and that impairment of mitochondrial homeostasis and the associated redox signaling may mediate the antineoplastic effect of the M arrest-inducing chemotherapeutics. Our findings provide insights into the fate of cells arrested at M phase and have implications in cancer therapy.

## Introduction

Cancer cells usually have high levels of reactive oxygen species (ROS) that can further drive cancer progression by activating or sustaining the oncogenesis [1, 2]. However, excessive production of ROS without commensurate increase in antioxidant defense may lead to cell death or senescence. Cancer cells have therefore generally acquired an enhanced antioxidant capacity to cope with the high output of ROS. Many commonly used cancer therapeutic agents are potent inducers of ROS production, and therapeutic strategies that are designed to disrupt the antioxidant defense system in cancer are also actively pursued [3, 4]. Interestingly, the tumor-suppressive effect caused by impaired DNA repair can also be mediated by increased oxidative stress [5, 6].

There appear to be many mechanisms by which cancer therapeutic agents can induce oxidative stress [2]. For example, ionizing radiation (IR) induces the production of mitochondrial superoxide [7]. Some agents are known to inhibit the function of NRF2, a master regulator of antioxidant defense [8]. PARP inhibitors can activate NADPH oxidases to produce more ROS [5, 9]. Interestingly, the oxidative stress induced by some anti-cancer agents was shown to correlate to or depend on cell cycle arrest at G2/M phases [10–13]. A mitochondrial link was implicated in IR-induced oxidative stress [11]. Etoposide was also found to drive an ATM-dependent mitochondrial biogenesis [14]. However, the function of the accumulated mitochondria in cells arrested at G2/M and their contribution to oxidative stress caused by cancer therapeutics remain to be characterized.

TH287 and TH588 were recently shown to exhibit potent antineoplastic activity. They function as dual inhibitor of MTH1, which sanitizes the oxidized dNTP pool [15], and tubulin polymerization [12, 16, 17]. They can induce G2/M arrest [16]. A recent study showed that it is the ROS buildup caused by mitotic arrest that contributes to the increased levels of 8-oxodGTP [13].

We here report how TH287 and paclitaxel can each induce oxidative stress that mediates their antineoplastic effects in fibrosarcoma cells HT1080 and osteosarcoma cells U2OS. We found that they both induced M arrest-coupled mitochondrial oxidative stress, and consequently exerted genotoxic effect and impaired cell proliferation. Although the mitochondrial mass was greatly increased in cells arrested at M phase, the mitochondria were functionally compromised. When cell cycle progression is decelerated by the inhibition or depletion of CHK1, pre-depletion of mitochondria, or the knockdown of PGC-1α, so that M arrest cannot be established, mitochondrial oxidative stress and the associated genotoxic effect caused by the cancer drugs are greatly alleviated. These findings establish that some cancer therapeutic agents exert their antineoplastic effect via inducing M arrest-coupled mitochondrial oxidative stress.

## Results

### TH287 and paclitaxel induce M arrest and mitochondrial oxidative stress

**Fig. 1:**
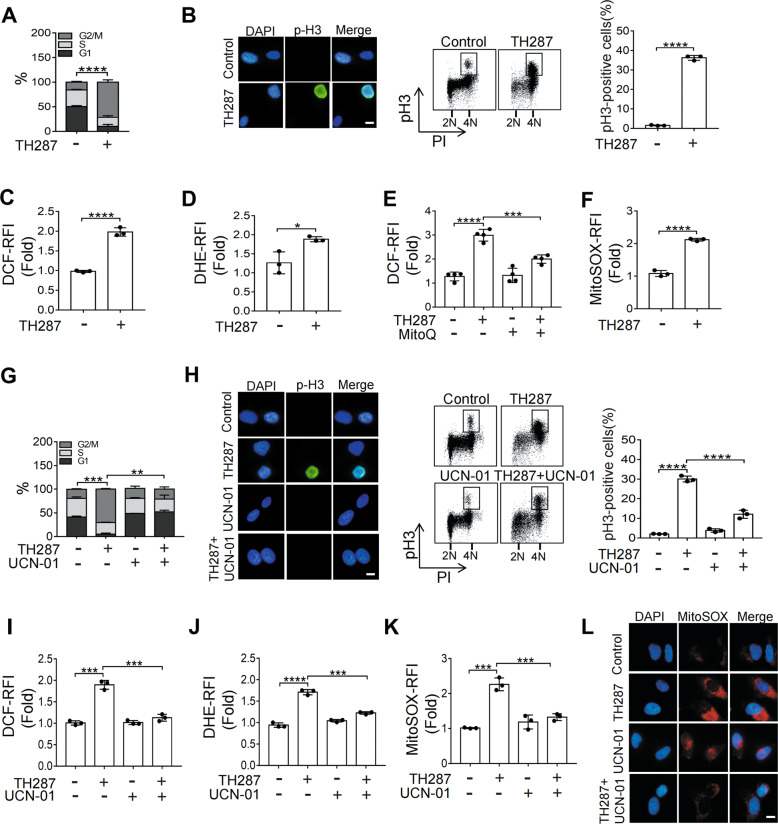
TH287 and paclitaxel induce M arrest and mitochondrial oxidative stress. **A** HT1080 cells were treated with DMSO or TH287(10 μM) for 24 h and then subjected to cell cycle distribution analysis by flow cytometry. The proportions of G2/M-phase cells were statistically analyzed. **B** Left, representative immunofluorescence imaging of phosphorylated Histone H3 (pH3) in HT1080 cells treated with DMSO or TH287 (10 μM) for 24 h. Scale bar, 5 μm. Right, representative cell-cycle profiles of HT1080 cells treated with DMSO or TH287 (10 μM) for 24 h assessed by pH3 (mitotic cells) and PI flow cytometric analysis (FACS). 2N DNA content indicates cells in G1 phase. 4 N DNA content indicates cells in either G2 or M phase. The mitotic index (percentage of pH3-positve cells) for each group was shown. **C** Flow cytometric analysis of intracellular ROS levels measured by DCFH-DA and dihydroethidium (DHE). HT1080 cells were treated with DMSO or TH287(10 μM) for 24 h. **D** Flow cytometric analysis of superoxide measured by dihydroethidium (DHE). HT1080 cells were treated with DMSO or TH287(10 μM) for 24 h. **E** Flow cytometric analysis of intracellular ROS levels measured by DCFH-DA. HT1080 were treated with TH287 (10 μM) alone or in combination with MitoQ (1 μM) for 24 h. **F** MitoSOX Red was used to assess mitochondrial superoxide levels. HT1080 cells were treated with DMSO or TH287(10 μM) for 24 h. Mitochondrial superoxide was measured using MitoSOX Red, detected by flow cytometric analysis. **G** HT1080 cells were pretreated with DMSO or UCN-01 (300 nM) for 4 h before addition of TH287 (10 μM) treatment for 24 h, and subjected to cell cycle distribution analysis by flow cytometry. The proportions of G2/M-phase cells were statistically analyzed. **H** Left, representative immunofluorescence imaging of pH3 in HT1080 cells treated with DMSO or UCN-01 (300 nM) for 4 h before addition of TH287 (10 μM) treatment for an additional 24 h. Scale bar, 5 μm. Right, representative cell-cycle profiles of HT1080 cells treated with DMSO or UCN-01 (300 nM) for 4 h before addition of TH287 (10 μM) treatment for an additional 24 h assessed by pH3/PI FACS. The mitotic index (percentage of pH3-positve cells) for each group was shown. **I** Flow cytometric analysis of intracellular ROS levels measured by DCFH-DA. HT1080 cells were treated with TH287 (10 μM) alone or in combination with UCN-01 (300 nM) for 24 h. **J** Flow cytometric analysis of superoxide measured by dihydroethidium (DHE). HT1080 cells were treated with TH287 (10 μM) alone or in combination with UCN-01 (300 nM) for 24 h. **K** HT1080 cells were treated with TH287 (10 μM) alone or in combination with UCN-01 (300 nM) for 24 h. Mitochondrial superoxide was detected by flow cytometric analysis after staining with MitoSOX Red. **L** HT1080 cells were treated with TH287 (10 μM) alone or in combination with UCN-01 (300 nM) for 24 h. Mitochondrial superoxide, indicated by MitoSOX Red, was detected using fluorescence microscope. Scale bar, 5 μm. Data shown were representative of three independent experiments and data presented in bars as mean ± S.D. The statistical differences between the two groups were analyzed by two-sided unpaired Student’s *t* test. *p < 0.05, **p < 0.01, ***p < 0.001, ****p < 0.0001.

TH287, which inhibits both MTH1 and tubulin polymerization, possesses high tumoricidal activity [15, 16, 18]. We examined the fates of cancer cells treated with TH287. As previously reported in HeLa cells [16], TH287 induced a striking G2/M arrest in three cancer cell lines tested, HT1080 (fibrosarcoma), U2OS (osteosarcoma), and MCF-7 (breast cancer) (Fig. 1A, Supplementary Fig. 1A and B). Further characterization of those cells by staining for the phosphorylation of histone H3 (p-H3) indicates that they are mostly arrested at M phase in all three cell lines tested (Fig. 1B, Supplementary Fig. 1C and D). The proportions of cells at M phase (p-H3 positive) were increased 10-fold or more (HT1080: from 1.5% to 36.2%, U2OS: from 2.4% to 21.7%, MCF-7: from 1.1% to 31.4%) (Fig. 1B, Supplementary Fig. 1C and D).

ROS were shown to peak in mitosis and prolonged mitotic arrest can exacerbate oxidative stress [10, 17]. TH588, another agent dually targeting MTH1 and tubulin, was shown to confer cancer cells oxidative stress in a mitotic arrest-dependent manner [13, 17]. Because TH287-treated cells were largely arrested at M phase, we speculated that the M arrest might lead to increased accumulation of ROS. We therefore measured the level of ROS in TH287-treated cells. Indeed, the intracellular level of ROS, as measured by DCFH-DA, was greatly increased by TH287 in HT1080 and U2OS cells (Fig. 1C, Supplementary Fig. 1E). Measurement of superoxide using dihydroethidium (DHE) showed a similar trend (Fig. 1D, Supplementary Fig. 1F). We next determined the source of ROS in TH287-treated cells. When mitoquinone (MitoQ), a mitochondria–targeted antioxidant, was applied to the cancer cells, the elevation of ROS caused by TH287 was greatly reduced (Fig. 1E), which suggests that the mitochondria may have contributed to ROS increase. We therefore measured the production of mitochondrial superoxide (O_2_^·−^) using MitoSOX Red, which emits red fluorescence when oxidized by mitochondrial O_2_^·−^. TH287 treatment resulted in an increased MitoSOX fluorescence intensity, as shown by flow cytometry analysis (Fig. 1F, Supplementary Fig. 1G).

Deletion or depletion of CHK1 can decrease mitotic index and abrogate M arrest [19–21]. We attempted to abolish the M arrest caused by TH287 by co-applying UCN-01, which is known to inhibit CHK1 and other kinases [22, 23]. When cancer cells were co-treated with TH287 and UCN-01, M arrest was largely abolished (Fig. 1G and H, Supplementary Fig. 1H and I). Correspondingly, ROS level remained unchanged when compared to control (Fig. 1I, Supplementary Fig. 1J). Measurement of superoxide using DHE showed a similar trend (Fig. 1J, Supplementary Fig. 1K). Importantly, TH287 failed to elevate MitoSOX in the presence of UCN-01, as measured by flow cytometry (Fig. 1K, Supplementary Fig. 1L). Examination of MitoSOX using a fluorescence microscope revealed the same trend (Fig. 1L, Supplementary Fig. 1M).

To further verify the role of CHK1 in establishing M arrest, we depleted CHK1 by RNAi in HT1080 cells. Depletion of CHK1 also abolished M arrest caused by TH287 (Supplementary Fig. 2A and B). Correspondingly, the levels of the total ROS and mitochondrial O_2_^·−^ remained unchanged in the CHK1 RNAi +TH287 group when compared to the control (Supplementary Fig. 2C and D).

Paclitaxel inhibits the disassembly of microtubules. We subjected HT1080 cells to paclitaxel for 24 h and analyzed the cell cycle distribution. The paclitaxel-treated cells also displayed M arrest (Supplementary Fig. 2E and F). Consistently, paclitaxel-induced M arrest was attenuated when cells were co-treated with UCN-01 (Supplementary Fig. 2E and F). When cells were treated with paclitaxel in combination with UCN-01, the ROS level was significantly reduced when compared to paclitaxel treatment alone (Supplementary Fig. 2G). Paclitaxel similarly resulted in an increased MitoSOX fluorescence intensity that could be attenuated by UCN-01 (Supplementary Fig. 2H, I and J). These results indicate that the induction of mitochondrial oxidative stress by the two antineoplastic agents is associated with M arrest.

### TH287 or paclitaxel induces M arrest-coupled mitochondrial accumulation

**Fig. 2:**
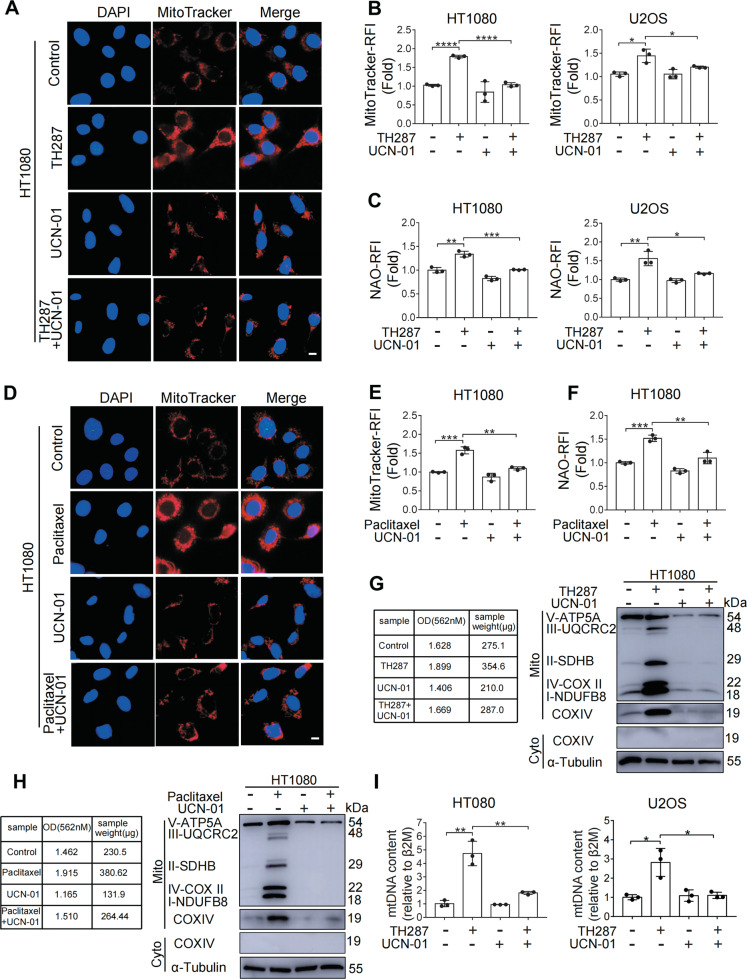
TH287 or paclitaxel induces M arrest-coupled mitochondrial accumulation. **A** Representative fluorescence imaging of mitochondria stained with MitoTracker Red in HT1080 cells treated with TH287 (10 μM) alone or in combination with UCN-01 (300 nM) for 24 h. Scale bar, 5 μm. **B** HT1080 and U2OS cells were treated with TH287 (10 μM) alone or in combination with UCN-01 (300 nM) for 24 h. Cells were stained with MitoTracker Red and analyzed using a flow cytometer. **C** HT1080 and U2OS cells were treated with TH287 (10 μM) alone or in combination with UCN-01 (300 nM) for 24 h. Cells were stained with nonylacridine orange (NAO) and analyzed using a flow cytometer. **D** Representative fluorescence imaging of mitochondria stained with MitoTracker Red in HT1080 cells treated with paclitaxel (50 nM) alone or in combination with UCN-01 (300 nM) for 24 h. Scale bar, 5 μm. **E** HT1080 cells were treated with paclitaxel (50 nM) alone or in combination with UCN-01 (300 nM) for 24 h. Cells were stained with MitoTracker Red and analyzed using a flow cytometer. **F** HT1080 cells were treated with paclitaxel (50 nM) alone or in combination with UCN-01 (300 nM) for 24 h. Cells were stained with nonylacridine orange (NAO) and analyzed using a flow cytometer. **G** HT1080 cells were treated with TH287 (10 μM) alone or in combination with UCN-01 (300 nM) for 24 h. Mitochondria fractions were isolated with the Cell Mitochondria Isolation Kit. The intact mitochondria were isolated from 20 million treated cells and mitochondrial proteins were quantified using BCA Protein Assay. Equal amounts of proteins were loaded in each lane. The mitochondrial nature of the isolated proteins was confirmed with Western blotting analysis using antibodies against various mitochondrial components. **H** HT1080 cells were treated with paclitaxel (50 nM) alone or in combination with UCN-01 (300 nM) for 24 h. Western blotting analysis using antibodies against various mitochondrial components were treated as described in **G**. **I** Mitochondrial DNA (mtDNA) copy number measured by quantitative PCR. The relative amounts of Cytochrome Oxidase I (COX I) in total DNA were determined as described in Materials and methods. Data shown were representative of three independent experiments and data presented in bars as mean ± S.D. The statistical differences between the two groups were analyzed by two-sided unpaired Student’s *t* test. *p < 0.05, **p < 0.01, ***p < 0.001, ****p < 0.0001.

We next determined whether the M arrest and the associated elevation in ROS might be associated with alterations in mitochondrial content. Staining of mitochondria with MitoTracker revealed a significantly increased mitochondrial content in TH287-treated cells (Fig. 2A and B). When cells were treated with TH287 and UCN-01 in combination, the increase in mitochondrial content could no longer be detected (Fig. 2A and B). Depletion of CHK1 abolished mitochondrial accumulation caused by TH287 (Supplementary Fig. 3A and B). Similarly, staining with nonylacridine orange (NAO), whose localization in mitochondria is independent of mitochondrial membrane potential (ΔΨm) and oxidation, also revealed more mitochondrial accumulation under TH287 treatment (Fig. 2C), which was significantly reduced when cells were treated with TH287 and UCN-01 in combination (Fig. 2C). Paclitaxel similarly induced M arrest-dependent mitochondrial accumulation (Fig. 2D, E and F). Depletion of CHK1 also abolished mitochondrial accumulation caused by paclitaxel (Supplementary Fig. 3C). We next measured the change in the amount of mitochondrial proteins that were isolated from same number of cells in each group with BCA Protein Assay. Interestingly, TH287-treated cells yielded more mitochondrial proteins than the control, but the amount of mitochondrial proteins remained at basal level when cells were treated with TH287 and UCN-01 in combination (Fig. 2G). Western blotting analysis confirmed the mitochondrial source of the isolated proteins (Fig. 2G, left). Interestingly, even though the same amount of proteins were loaded in each lane, more mitochondrial components were detected in TH287 treatment group (Fig. 2G, right), suggesting that mitochondrial proteins could be more readily isolated and were thus more abundantly enriched in TH287-treated cells, even under the same procedure. Paclitaxel similarly resulted in increased accumulation of mitochondrial components that could be attenuated by UCN-01 (Fig. 2H). Depletion of CHK1 by RNAi also attenuated mitochondrial accumulation caused by TH287 or paclitaxel (Supplementary Fig. 3D and E). Mitochondrial DNA (mtDNA) was detected in higher copy numbers in cells treated with TH287 than in control, but remained at basal level or lower in the co-treatment group (Fig. 2I). In addition, the increase in mtDNA caused by TH287 was also attenuated by CHK1 depletion (Supplementary Fig. 3F). Together, these results indicate that TH287 and paclitaxel can each cause M-arrest coupled mitochondrial accumulation.

### Mitochondrial function is impaired in cells arrested at M phase

**Fig. 3:**
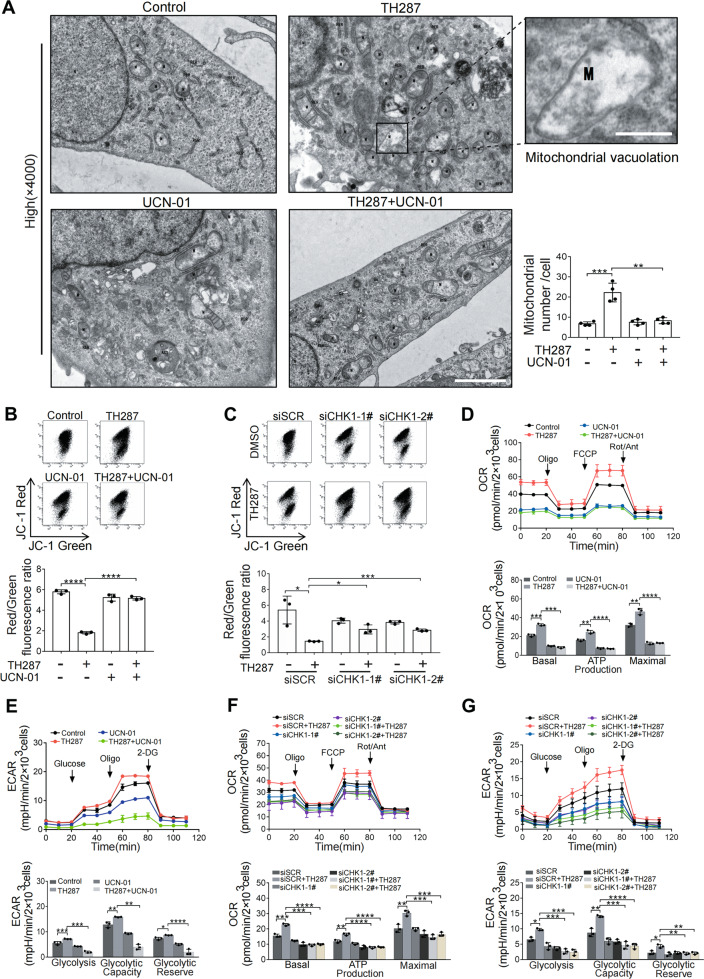
Mitochondrial function is impaired in cells arrested at M phase. **A** Transmission electron micrographs of HT1080 cells treated with TH287 (10 μM) alone or in combination with UCN-01 (300 nM) for 24 h. There are more mitochondria and Mitochondrial vacuolation are apparent in TH287-treated cells. Magnifications: high power lens, ×4000, Scale bar, 2.0 μM. N, Nucleus. M, Mitochondria. REF, Rough endoplasmic reticulum. Go, Golgi apparatus. ASS, Autolysosome. Boxes represent the areas seen in the high-power-magnification images. Scale bar, 0.5 μM. Quantitative analysis of number of mitochondria in HT1080 cells treated with TH287 and/or UCN-01. **B** HT1080 cells treated with TH287 (10 μM) alone or in combination with UCN-01 (300 nM) for 24 h were stained with JC-1 according to Materials and methods and analyzed using a flow cytometer. Red fluorescence represents the mitochondrial aggregate form of JC-1 (JC-1 polymers), which indicates the intact mitochondrial membrane potential. Green fluorescence represents the monomeric form of JC-1 (JC-1 monomers), which indicates the dissipation of mitochondrial transmembrane potential. Ratios of JC-1 monomer to JC-1 polymer (red/green fluorescence) was calculated. **C** HT1080 cells were transfected with non-targeted control siRNA or CHK1-directed siRNA for 48 h. Subsequently, cells were treated with 10 μM TH287 for 24 h and then were treated as described in **B**. Kinetic profile of ECAR and OCR in HT1080 cells was measured in real-time, under basal conditions and in response to mitochondrial drugs oligomycin, FCCP, Rotenone & antimycin A for OCR (**D**), and in response to glucose, oligomycin and 2-DG for ECAR (**E).** Indices of mitochondrial respiratory function, calculated from OCR profile: basal OCR, ATP-linked OCR and maximal OCR (see also Materials and Methods section). Indices of glycolytic pathway activation, calculated from ECAR profile: glycolysis, glycolytic capacity and glycolytic reserve (see also Materials and Methods section). **F** and **G** Kinetic profile of ECAR and OCR in HT1080 cells transfected with with non-targeted control siRNA or CHK1-directed siRNA was measured as described in **D** and **E**. Data shown were representative of three independent experiments and data presented in bars as mean ± S.D. The statistical differences between the two groups were analyzed by two-sided unpaired Student’s *t* test. *p < 0.05, **p < 0.01, ***p < 0.001, ****p < 0.0001.

We next evaluated the function of the accumulated mitochondria in TH287-treated cells. We first directly visualized the cellular contents using transmission electron microscopy. There were more mitochondria in TH287-treated HT1080 than in control cells, and vacuolation was apparent in some mitochondria. However, when cells were simultaneously treated with TH287 and UCN-01 to block their entry into M phase, the mitochondrial accumulation did not occur (Fig. 3A). JC-1 can aggregate in normal mitochondria and emit red fluorescence, while mitochondria with loss of mitochondrial membrane potential (MMP) would emit green fluorescence, and the ratio of red to green fluorescence reflects the integrity of MMP. We observed that while TH287 significantly decreased the red to green fluorescence ratio, reflecting the loss of MMP, the decrease was attenuated in the TH287 and UCN-01 co-treatment group (Fig. 3B). MMP restorations were also obtained with TH287 and CHK1 RNAi co-treatment, though to a lesser extent (Fig. 3C). The activity of numerous proteins in mitochondria is modulated by reversible acetylation during various metabolic stresses [24–26]. A recent study showed that SUMOylation occurring inside mitochondria could affect the acetylation of mitochondrial proteins [27]. We detected an increased level of SUMO-conjugated proteins in the mitochondrial fraction of the TH287 treatment group (Supplementary Fig. 4A). Importantly, these SUMO-conjugated proteins were significantly reduced in amount when cells were treated by TH287 and UCN-01 in combination. Paclitaxel similarly resulted in more mitochondrial SUMOylation that could be attenuated by UCN-01 (Supplementary Fig. 4B). We next examined the acetylation level of mitochondrial proteins. As shown in Supplementary Fig. 4C and D, the global acetylation of mitochondrial proteins was markedly increased in TH287 or paclitaxel-treated cells. The increase was again associated with M arrest. We then measured extracellular acidification rate (ECAR) and oxygen consumption rate (OCR) that were associated with mitochondrial function. While the levels of basal and maximal OCR were elevated by TH287, they were significantly reduced by UCN-01 (Fig. 3D), suggesting a reduction in the mitochondrial respiration in the presence of UCN-01. It should be noted that UCN-01 treatment alone reduced the OCR (Fig. 3D) and the total amount of mitochondrial proteins (Fig. 2G and H). Furthermore, ECAR analysis showed a higher glycolytic capacity in TH287-treated cells (Fig. 3E), indicating that the increased mitochondrial mass did not suffice the bioenergertic need in the cells arrested at M phase. Similar results were obtained with cells simultaneously subjected to CHK1 RNAi and TH287 treatment (Fig. 3F and G). These dysfunctional mitochondria resemble those in senescent cells which solely rely on glycolysis for ATP production despite a greatly increased mitochondrial mass [28]. Together, these data indicate that mitochondria that accumulate in M-arrested cells are functionally compromised.

### Pre-depletion of mitochondria attenuates M arrest-dependent oxidative stress

**Fig. 4:**
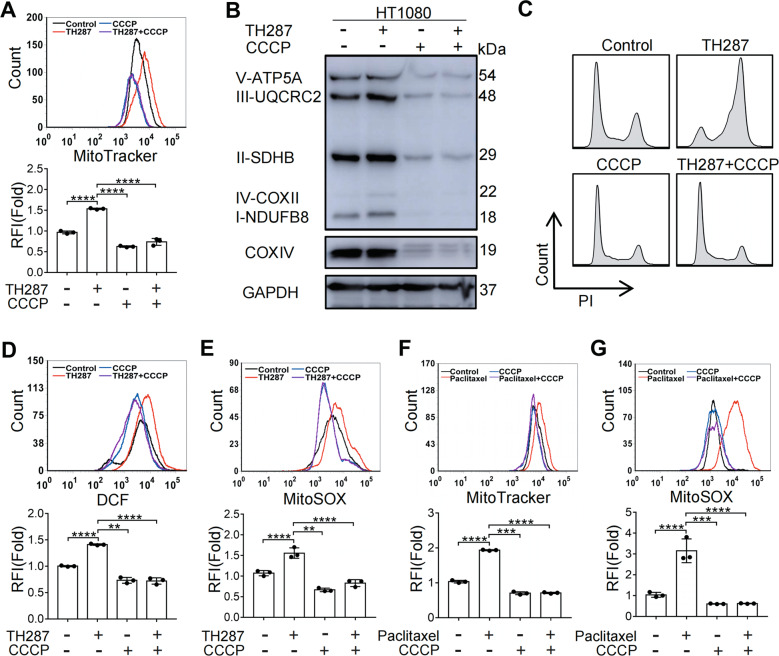
Pre-depletion of mitochondria attenuates M arrest-dependent oxidative stress. **A** Flow cytometry analysis and quantification of mitochondrial mass in HT1080 cells pretreated with CCCP (12.5 μM) for 48 h, followed by treatment with TH287 (10 μM) for 24 h. **B** Western blotting analysis of OXPHOS complex and COXIV protein levels in HT1080 cells pretreated with CCCP (12.5 μM) for 48 h before TH287 treatment for 24 h. **C** HT1080 cells pretreated with CCCP (12.5 μM) for 48 h before TH287 treatment for 24 h and were then subjected to cell cycle distribution analysis by flow cytometry. **D** Flow cytometric analysis of intracellular ROS levels measured by DCFH-DA. HT1080 were pretreated with CCCP (12.5 μM) for 48 h before TH287 treatment for 24 h. **E** Flow cytometric analysis of Mitochondrial superoxide levels by MitoSOX Red in HT1080 cells pretreated with CCCP (12.5 μM) for 48 h, followed by treatment with TH287 (10 μM) for 24 h. **F** Flow cytometry analysis and quantification of mitochondrial mass in HT1080 cells pretreated with CCCP (12.5 μM) for 48 h, followed by treatment with paclitaxel (50 nM) for 24 h. **G** Flow cytometric analysis of Mitochondrial superoxide levels by MitoSOX Red in HT1080 cells pretreated with CCCP (12.5 μM) for 48 h, followed by treatment with paclitaxel (50 nM) for 24 h. Data shown were representative of three independent experiments and data presented in bars as mean ± S.D. The statistical differences between the two groups were analyzed by two-sided unpaired Student’s *t* test. **p < 0.01, ***p < 0.001, ****p < 0.0001.

The data shown above indicate that CHK1 functional disruption decelerates cell cycle, spares the M arrest and attenuates mitochondrial accumulation and the associated oxidative stress. To further confirm mitochondrial stress as a mediator of the cancer drugs, we pre-depleted mitochondria in HT1080 cells with carbonyl cyanide 3-chlorophenylhydrazone (CCCP) [28], which causes the dissipation of MMP and consequently mitophagy, and then subjected the cells to TH287 treatment. This attempt was intended to reduce mitochondrial accumulation and alleviate mitochondrial stress at M phase. As shown in Fig. 4A and B, when cells were first treated with CCCP and then with TH287, mitochondrial accumulation was indeed abolished. However, the CCCP treatment did not just deplete mitochondria but also abolished the induction of M arrest (Fig. 4C). As expected, the levels of ROS and MitoSOX were commensurate with the mitochondrial contents in CCCP-treated cells (Fig. 4D and E). Similarly, the pre-treatment with CCCP precluded the accumulation of mitochondria and superoxide caused by paclitaxel (Fig. 4F and G). These data indicate that functional mitochondria probably need to be in sufficient supply for the cancer cells to enter mitosis, and it could be difficult to obtain mitotic cells with reduced mitochondrial content.

### M arrest is coupled with increased mitochondrial biogenesis

**Fig. 5:**
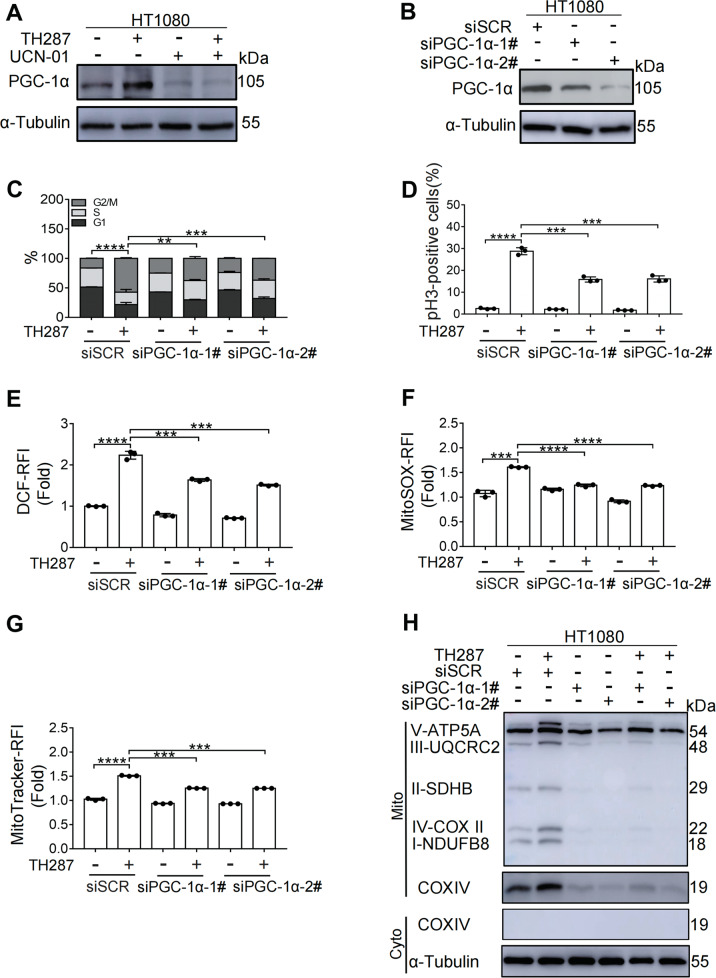
M arrest is coupled with increased mitochondrial biogenesis. **A** Western blotting analysis of PGC-1α protein levels in HT1080 cells treated with TH287 (10 μM) alone or TH287 in combination with UCN-01 (300 nM) for 24 h. **B** HT1080 cells were transfected with non-targeted control siRNA or PGC-1α-directed siRNA for 48 h. Western blotting analysis the RNAi efficiency of PGC-1α in HT1080 cells. **C** HT1080 cells were transfected with non-targeted control siRNA or PGC-1α-directed siRNA for 48 h. Subsequently, cells were treated with 10 μM TH287 for 24 h, and then subjected to cell cycle distribution analysis by flow cytometry. The proportions of G2/M-phase cells were statistically analyzed. **D** HT1080 cells were transfected with non-targeted control siRNA or PGC-1α-directed siRNA for 48 h. Subsequently, cells were treated with 10 μM TH287 for 24 h and mitotic index was assessed by pH3/PI FACS. The mitotic indices were statistically analyzed. **E** HT1080 cells were transfected with non-targeted control siRNA or PGC-1α-directed siRNA for 48 h. Flow cytometric analysis of intracellular ROS levels measured by DCFH-DA in HT1080-siSCR and HT1080-siPGC-1α cells treated with TH287 (10 μM) for 24 h. **F** HT1080 cells were transfected with non-targeted control siRNA or PGC-1α-directed siRNA for 48 h. Flow cytometric analysis of Mitochondrial superoxide levels by MitoSOX Red in HT1080-siSCR and HT1080-siPGC-1α cells treated with TH287 (10 μM) for 24 h. **G** HT1080 cells were transfected with non-targeted control siRNA or siPGC-1α-directed siRNA for 48 h. Subsequently, cells were treated with 10 μM TH287 for 24 h and were stained with MitoTracker Red and analyzed using a flow cytometer. **H** HT1080 cells were transfected with non-targeted control siRNA or siPGC-1α-directed siRNA for 48 h. Subsequently, cells were treated with 10 μM TH287 for 24 h and Mitochondria fractions were isolated. The mitochondrial nature of the isolated proteins was confirmed with Western blotting analysis using antibodies against various mitochondrial components. Data shown were representative of three independent experiments and data presented in bars as mean ± S.D. The statistical differences between the two groups were analyzed by two-sided unpaired Student’s *t* test. **p < 0.01, ***p < 0.001, ****p < 0.0001.

PGC-1α is a key regulator of mitochondrial biogenesis and oxidative metabolism [29, 30]. To determine whether PGC-1α is involved in TH287-induced mitochondrial accumulation, we examined the expression of PGC-1α in HT1080 cells treated with TH287 and found that the protein level of PGC-1α was significantly increased (Fig. 5A). Consistently, cells treated with paclitaxel also displayed an increased level of PGC-1α (Supplementary Fig. 5A). We next determined whether depletion of PGC-1α, and consequently a blockade of mitochondrial biogenesis, would alleviate mitochondrial stress. Unexpectedly, when cells were depleted of PGC-1α by RNAi, their cell cycle progression was also decelerated, resulting in fewer cells arrested at M phase in response to TH287 or paclitaxel (Fig. 5B, C, D and Supplementary Fig. 5B). Consistently, the elevation of ROS caused by TH287 was significantly attenuated by PGC-1α depletion (Fig. 5E). In addition, mitochondrial O_2_^·−^ induction by TH287 was also attenuated by PGC-1α depletion (Fig. 5F). As expected, depletion of PGC-1α by RNAi attenuated mitochondrial accumulation (Fig. 5G and H). Similar results were obtained with cells simultaneously subjected to PGC-1α RNAi and paclitaxel treatment (Supplementary Fig. 5). These data further support that sufficient supply of mitochondria is required for the induction of M arrest by the two anti-cancer agents.

### Mitochondrial insufficiency is associated with decelerated cell cycle progression

**Fig. 6:**
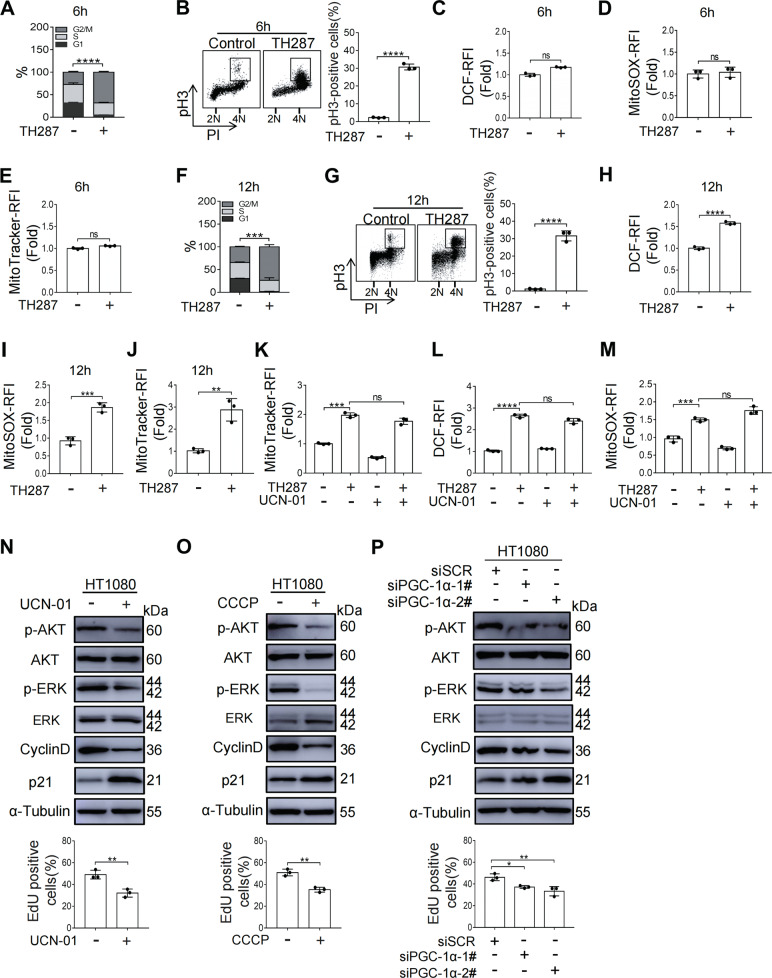
Mitochondrial insufficiency is associated with decelerated cell cycle progression. **A** HT1080 cells were treated with DMSO or TH287 (10 μM) for 6 h and then subjected to cell cycle distribution analysis by flow cytometry. The proportions of G2/M-phase cells were statistically analyzed. **B** HT1080 cells were treated with DMSO or TH287 (10 μM) for 6 h and subjected to pH3/PI FACS. The mitotic indices were statistically analyzed. **C** Flow cytometric analysis of intracellular ROS levels measured by DCFH-DA. HT1080 cells were treated with TH287 (10 μM) for 6 h. **D** Flow cytometric analysis of Mitochondrial superoxide levels by MitoSOX Red in HT1080 cells treated with TH287 (10 μM) for 6 h. **E** Mitochondrial mass measured by MitoTracker Red staining. HT1080 cells were treated with TH287 (10 μM) for 6 h. **F** HT1080 cells were treated with DMSO or TH287 (10 μM) for 12 h and then subjected to cell cycle distribution analysis by flow cytometry. The proportions of G2/M-phase cells were statistically analyzed. **G** HT1080 cells were treated with DMSO or TH287 (10 μM) for 12 h and subjected to pH3/PI FACS. The mitotic indices were statistically analyzed. **H** Flow cytometric analysis of intracellular ROS levels measured by DCFH-DA. HT1080 cells were treated with TH287 (10 μM) for 12 h. **I** Flow cytometric analysis of Mitochondrial superoxide levels by MitoSOX Red in HT1080 cells treated with TH287 (10 μM) for 12 h. **J** Mitochondrial mass measured by MitoTracker Red staining. HT1080 cells were treated with TH287 (10 μM) for 12 h. **K** Flow cytometry analysis and quantification of mitochondrial mass in HT1080 cells pretreated with TH287 (10 μM) for 12 h, followed by treatment with UCN-01 (300 nM) for 24 h. **L** Flow cytometric analysis of intracellular ROS levels measured by DCFH-DA. HT1080 cells were pretreated with TH287 (10 μM) for 12 h, followed by treatment with UCN-01 (300 nM) for 24 h. **M** Flow cytometric analysis of Mitochondrial superoxide levels by MitoSOX Red in HT1080 cells pretreated with TH287 (10 μM) for 12 h, followed by treatment with UCN-01 (300 nM) for 24 h. **N** Western blotting analysis of p-AKT, total AKT, p-ERK, total ERK, CyclinD and p21 protein levels in HT1080 cells treated with UCN-01 (300 nM) for 24 h and Quantification of EdU-positive cells after 300 nM UCN-01 treatment for 24 h. **O** Western blotting analysis of p-AKT, total AKT, p-ERK, total ERK, CyclinD and p21 protein levels in HT1080 cells treated with CCCP (12.5 μM) for 48 h and Quantification of EdU-positive cells after 12.5 μM CCCP treatment for 48 h. **P** Western blotting analysis of p-AKT, total AKT, p-ERK, total ERK, CyclinD and p21 protein levels in HT1080 cells transfected with non-targeted control siRNA or PGC-1α-directed siRNA for 48 h and Quantification of EdU-positive cells after the depletion of PGC-1α by RNAi for 48 h. Data shown were representative of three independent experiments and data presented in bars as mean ± S.D. The statistical differences between the two groups were analyzed by two-sided unpaired Student’s *t* test. *p < 0.05, **p < 0.01, ***p < 0.001, ****p < 0.0001, ns stand for no significant.

We next determined the sequential occurrences of M arrest and mitochondrial accumulation after TH287 treatment. While TH287 treatment for 6 h could also result in M arrest (30.7% at M phase vs. 2.3% in control) (Fig. 6A and B), the levels of ROS, mitochondrial superoxide (O_2_^·−^) and mitochondrial content were not found to be increased at this stage (Fig. 6C, D and E). These data suggest that the M arrest occurs prior to the emergence of mitochondrial accumulation and the associated increase of mitochondrial ROS.

We next tested whether mitochondrial homeostasis could still be restored by UCN-01 after the mitochondrial stress had occurred. To this end, HT1080 cells were first treated with TH287 for 12 h, which also resulted in M arrest and increased the proportion of cells in mitosis (36.0% vs. 1.5% in control) (Fig. 6F and G). The levels of intracellular ROS and mitochondrial superoxide, as measured by DCFH-DA and MitoSOX, respectively, were greatly increased by TH287 treatment for 12 h (Fig. 6H and I). Correspondingly, such treatment led to a significantly increased mitochondrial accumulation, as shown by MitoTracker staining (Fig. 6J). We observed that UCN-01 treatment was not effective in reducing mitochondrial content when being added after the mitochondrial accumulation had occurred (Fig. 6K). Moreover, the levels of ROS and MitoSOX caused by TH287 treatment remained high (Fig. 6L and M). These results suggest that UCN-01 may function to block the accumulation of dysfunctional mitochondria by decelerating cell cycle progression that is required for the initiation of M arrest, rather than by promoting the clearance of the accumulated mitochondria.

To substantiate the notion that a deceleration of cell cycle may protect the cells from the cytotoxic effect of TH287 or paclitaxel by blocking their entry into M phase, we measured the protein levels of genes that regulate cell cycle. When we separately treated cells with UCN-01, CCCP, and depletion of PGC-1α by RNAi,we observed reductions in cyclin D, p-AKT, and p-ERK, but an increased level of p21 (Fig. 6N, O and P). Consistently, the percentage of ethynyl-2′-deoxyuridine (EdU) positive cells was also decreased (Fig. 6N, O and P). These data indicate that cell cycle progression is indeed decelerated by each of those treatments.

### Abrogation of M arrest reduces genotoxicity of cancer drugs

**Fig. 7:**
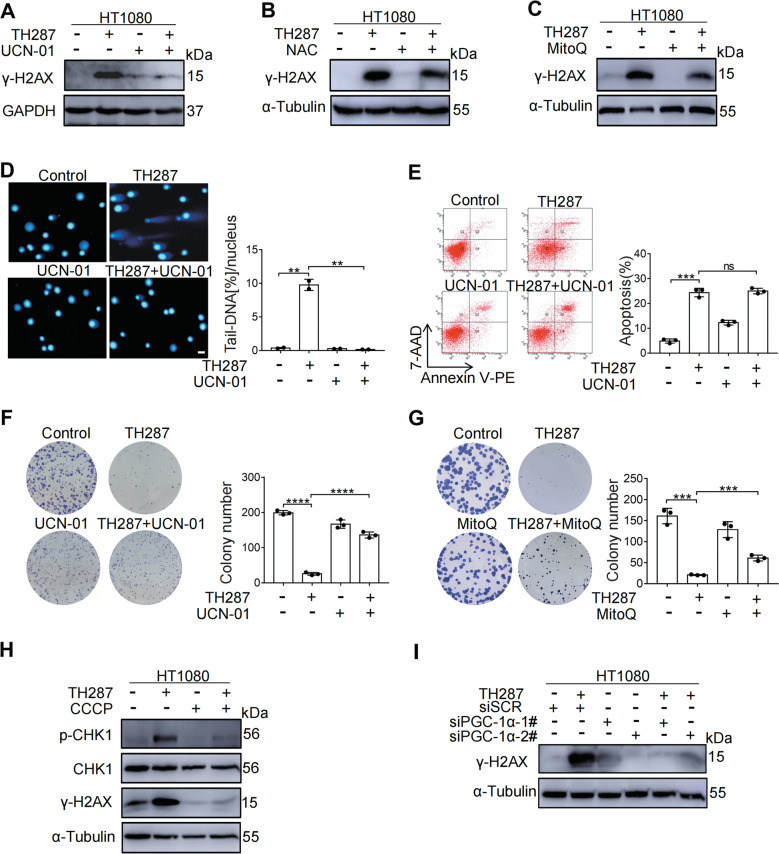
Cell cycle deceleration attenuates genotoxicity associated with M arrest. **A** Western blotting analysis of γ-H2AX protein levels in HT1080 cells treated with TH287 (10 μM) alone or TH287 in combination with UCN-01 (300 nM) for 24 h. **B** Western blotting analysis of γ-H2AX protein levels in HT1080 cells treated with TH287 (10 μM) alone or TH287 in combination with NAC (10 mM) for 24 h. **C** Western blotting analysis of γ-H2AX protein levels in HT1080 cells treated with TH287 (10 μM) alone or TH287 in combination with MitoQ (1 μM) for 24 h. **D** The amount of DNA strand breaks was quantified by measuring the amount of tail-DNA using the neutral comet assay as described in methods. HT1080 cells treated with TH287 (10 μM) alone or in combination with UCN-01 (300 nM) for 24 h. Representative pictures were shown on the left. Values represent average ± S.D. from two independent experiments (≥100 comets per experiment). Scale bar, 25 μm. **E** Scatterplots of apoptotic cells. Cells were treated with TH287 (10 μM) alone or in combination with UCN-01 (300 nM) for 24 h, and then harvested for analysis of apoptosis using Annexin V PE Apoptosis Detection Kit I. **F** Clonogenic assay of HT1080 cells treated with TH287 (10 μM) alone or in combination with UCN-01 (300 nM). **G** Clonogenic assay of HT1080 cells treated with TH287 (10 μM) alone or in combination with MitoQ (1 μM). **H** Western blotting analysis of p-CHK1, total CHK1 and γ-H2AX protein levels in HT1080 cells pretreated with CCCP (12.5 μM) for 48 h before TH287 (10 μM) treatment for 24 h. **I** HT1080 cells were transfected with non-targeted control siRNA or siPGC-1α-directed siRNA for 48 h. Western blotting analysis of γ-H2AX protein levels in HT1080-siSCR and HT1080-siPGC-1α cells treated with TH287 (10 μM) for 24 h. Data shown were representative of three independent experiments and data presented in bars as mean ± S.D. The statistical differences between the two groups were analyzed by two-sided unpaired Student’s *t* test. **p < 0.01, ***p < 0.001, ****p < 0.0001, ns stand for no significant.

Because administration of UCN-01 could spare M arrest and the associated oxidative stress, we next tested if it could also alleviate oxidative DNA damage and increase the survival and proliferation of cancer cells treated with TH287. Western blotting analysis of γ-H2AX, a marker of DNA double-strand breaks, confirmed that UCN-01 could reduce the level of DNA damage induced by TH287 (Fig. 7A). As expected, antioxidant N-acetylcysteine (NAC) significantly attenuated the induction of DNA damage by TH287 (Fig. 7B). Furthermore, when cells were treated with TH287 and mitoquinone (MitoQ) in combination, the DNA damage signaling was attenuated (Fig. 7C). The induction of DNA damage by TH287 was further confirmed using neutral comet assay, as evidenced by the presence of long comet tails (Fig. 7D). However, when cells were treated with TH287 in combination with UCN-01, the comet tailing was significantly reduced (Fig. 7D). CHK1 was reported to suppress apoptotic cell death [31]. To determine whether the reduction of DNA damage was due to disappearance of cells with damaged DNA, we evaluated the level of apoptosis by flow cytometry. We found that while TH287 and UCN-01 can each induce apoptosis, there was no additive effect when the two were used in combination (Fig. 7E). Importantly, clonogenic assay showed that both UCN-01 and MitoQ significantly rescued the impaired proliferation of TH287-treated cancer cells (Fig. 7F and G).

Paclitaxel similarly resulted in an increased the level of γ-H2AX that could be attenuated by UCN-01 or MitoQ (Supplementary Fig. 6A and B) and rescued the impaired proliferation of paclitaxel-treated cancer cells (Supplementary Fig. 6C and D).

Pre-treatment with CCCP precluded the accumulation of mitochondria and superoxide caused by TH287 or paclitaxel. It also greatly attenuated CHK1 activation (Fig. 7H and Supplementary Fig. 6E). Importantly, DNA damage, as reflected by the level of γ-H2AX, caused by TH287 or paclitaxel was greatly attenuated by CCCP (Fig. 7H and Supplementary Fig. 6E). Consistently, the levels of phosphorylated CHK1 and γ-H2AX confirmed that the depletion of PGC-1α by RNAi could reduce the level of DNA damage induced by TH287(Fig. 7I). These results indicate that the increased oxidative stress caused by TH287 or paclitaxel may impair cell proliferation by inflicting DNA damage.

### UCN-01 attenuates the antitumor effect of TH287 in vivo

**Fig. 8:**
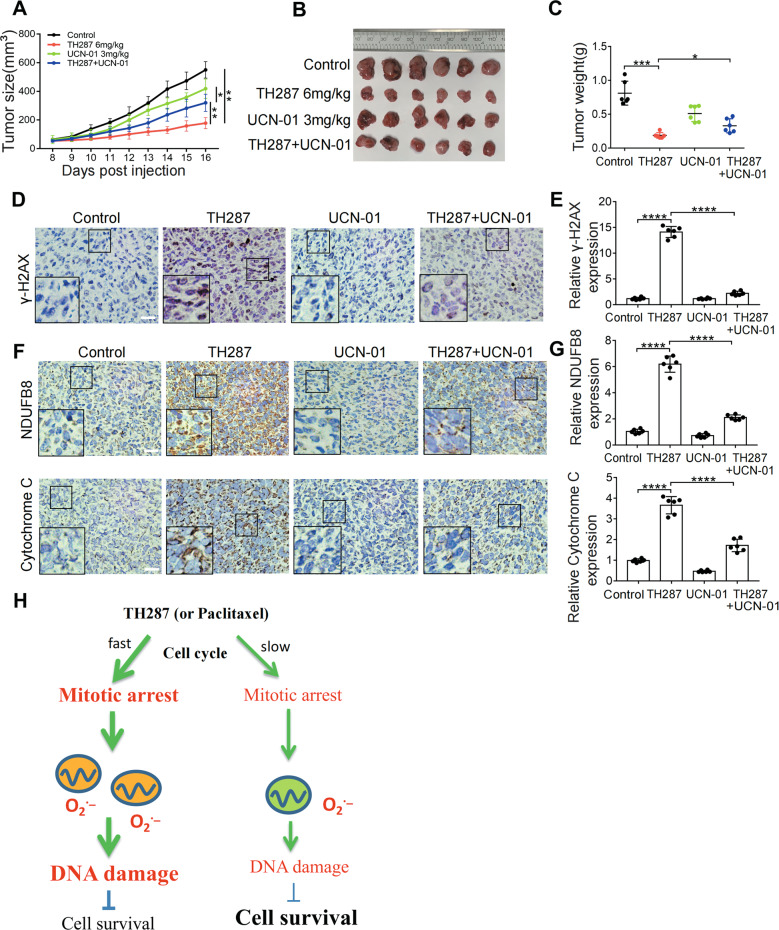
UCN-01 attenuates the antitumor effect of TH287 in vivo. **A**, **B**, **C** Growth curves and final weights of tumors from transplanted HT1080 cells in nude mice. Mice were randomized into one of four groups; vehicle only (*n* = 6), 6 mg/kg TH287 only (*n* = 6), 3 mg/kg UCN-01 only (*n* = 6) or 6 mg/kg TH287 plus 3 mg/kg UCN-01 (*n* = 6). Final weights were taken at day 16. **D** Representative IHC images showing the γ-H2AX. Scale bar, 20 μm. **E** Quantification of the relative intensity of IHC staining of γ-H2AX was performed using ImageJ software. **F** Representative IHC images showing the NDUFB8 and Cytochrome C. Scale bar, 20 μm. **G** Quantification of the relative intensity of IHC staining of NDUFB8 and Cytochrome C was performed using ImageJ software. **H** A schematic model. When cell cycle is decelerated, via the inhibition of CHK1, pre-depletion of mitochondria, or blocking mitochondrial biogenesis by PGC-1α depletion, so that M-arrest is spared or attenuated, mitochondria will be more likely to stay homeostatic, thus generating less ROS and rendering cancer cells less responsive to the therapeutic agents. Data shown were representative of three independent experiments and data presented in bars as mean ± S.D. The statistical differences between the two groups were analyzed by two-sided unpaired Student’s *t* test and ANOVA was used to compare significant differences among multiple experimental groups. *p < 0.05, **p < 0.01, ****p < 0.0001.

We next tested whether M arrest is required for the antitumor effect of TH287 in vivo. To this end, we subcutaneously inoculated HT1080 cells into nude mice. Eight days later, TH287 and UCN-01 were administered alone or in combination via peritoneal injection. We found that while TH287 greatly inhibited the growth of tumor xenografts, its tumor-suppressive effect was significantly attenuated by UCN-01 (Fig. 8A–C). Immunohistochemistry showed that the level of γ-H2AX was significantly increased by TH287 treatment, but was reduced by UCN-01 (Fig. 8D and E). Consistent with TH287-induced mitochondrial accumulation in cultured HT1080 cells, the levels of mitochondrial components NDUFB8 and Cytochrome C, as examined by immunohistochemistry, were also increased in tumor grafts treated with TH287. Importantly, UCN-01 also reduced the mitochondrial mass induced by TH287 treatment in vivo (Fig. 8F and G). These results confirm that mitochondrial accumulation also occurs in vivo in response to TH287 treatment, but can also be abolished by UCN-01.

## Discussion

Many cancer therapeutic agents arrest cells at M phase. Microtubule-targeting agents, which include paclitaxel and are commonly used in the treatment of ovarian, breast and lung cancer, are believed to trigger mitotic arrest by interfering with spindle assembly and disassembly. However, mitotic arrest does not necessarily lead to cell death because cells that escape the mitotic arrest (mitotic slippage) may survive as tetraploid cells and continue to divide. Activation of mitotic checkpoint, which is required for mitotic arrest, has been reported to either promote or weaken cell killing [32]. How the activation of checkpoint affects the fate of arrested cells appears to depend on numerous factors. We here show that cancer cells arrested at M phase are overwhelmed by the accumulating mitochondria that are functionally compromised but can produce more ROS, a condition that can be referred to as disruption of mitochondrial homeostasis. This mitochondrial stress thus functions as a mediator of the antineoplastic effect of the agents that cause M arrest. When cell cycle is decelerated, via the treatment with UCN-01, the depletion of CHK1, pre-depletion of mitochondria, or blocking mitochondrial biogenesis by PGC-1α depletion, so that M-arrest is spared or attenuated, mitochondria will be more likely to stay homeostatic, thus generating less ROS and rendering cancer cells less responsive to the therapeutic agents. Together, these results support a causal link between M arrest and a disrupted mitochondrial homeostasis and establish mitochondrial stress as a critical mediator of some cancer therapeutic agents.

ROS level fluctuates during the cell cycle and is associated with stage-specific cellular functions [33]. A recent study showed that ROS peak in mitosis and prolonged mitotic arrest can further increase the ROS level and cause more oxidative damage to biomolecules [10]. Cells arrested at G2/M phase in response to ionizing radiation have been shown to harbor more mitochondria and exhibit a higher level of oxidative stress [11]. As a dual inhibitor of tubulin polymerization and MTH1, TH287 is not supposed to increase the intracellular ROS level directly. The prolonged M arrest, however, may have allowed the accumulation of mitochondrial ROS that can increasingly inflict DNA damage. Although the antitumor effect of TH287 may be primarily mediated by its inhibition of tubulin, it is possible that its inhibition of MTH1 may also contribute to the induction of DNA damage when cells become more dependent on MTH1 with increased oxidative stress.

CHK1 is a key checkpoint protein that integrates signals from ATM and ATR [34, 35]. CHK1 activity has been shown to play a role in cell cycle checkpoints including G1-S, intra-S phase, G2/M, and mitotic spindle checkpoints [19, 36]. CHK1 acts as an essential effector that arrest cells at G2/M phase in response to genotoxic stress and oxidative stress [37–45]. Not only functioning to arrest cell cycle progression, CHK1 can also enhance homologous recombination repair via direct phosphorylation of RAD51 [46, 47]. It was also reported to possess an anti-apoptotic function [31]. Because of the apparently essential function of CHK1 for cell survival [41], CHK1 inhibitors were widely explored for their potential cancer therapeutic effects as a monotherapy or in combination with other cancer drugs [35, 48–51]. On the other hand, there are reports showing that CHK1 activation is required for the anticancer effect of many chemotherapeutic drugs. For example, CHK1-deficient cells fail to sustain mitotic arrest in the presence of taxol and checkpoint failure is associated with decreased Aurora-B activity and defects in phosphorylation and localization of BubR1 to kinetochores [19]. Our results indicate that UCN-01 or CHK1 depletion can protect cancer cells from agents that induce M arrest and the associated mitochondrial stress. It appears that either treatment benefits the cancer cells not by directly reducing the mitochondria in excess, but by decelerating cell cycle progression.

We further observed that when mitochondria were pre-depleted, the genotoxic effect of TH287 or paclitaxel was greatly attenuated, indicating that mitochondrial stress contributes substantially to the antineoplastic effect of these agents. Mitochondrial biogenesis is essential for many physiological processes [52]. However, aberrant activation of mitochondrial biogenesis and the associated induction of oxidative stress can lead to accelerated depletion of hematopoietic stem cells and thus impair hematopoiesis [53]. Mitochondria were reported to increasingly accumulate in cells exposed to DNA-damaging agents [11, 14, 28] and in cancer cells deficient in DNA repair [6]. Mitochondrial biogenesis actually promotes cellular senescence and is a key determinant of some phenotypes of senescent cells [28, 54]. Depletion of mitochondria in senescent cells resulted in no reduction in ATP production [28], suggesting that the mitochondria in excessive presence during the induction of senescence are not for fulfilling bioenergetic need, but rather a manifestation of disrupted mitochondrial homeostasis. Disruption of mitochondrial homeostasis similarly occurs in ovarian cancer cells in which RAD51 is depleted [6]. Increased acetylation and SUMOylation of mitochondrial proteins are characteristic of impaired mitochondrial bioenergetics [24–27]. We observed that the levels of acetylation and SUMOylation of mitochondrial proteins were significantly increased in cancer cells that were arrested at M phase. The disruption of mitochondrial homeostasis and the exacerbation of oxidative stress by the dysfunctional mitochondria may have ultimately led to the demise of cancer cells.

We intended to alleviate the mitochondrial stress associated with M arrest by accelerating the clearance of mitochondria or by blocking mitochondrial biogenesis. However, either treatment decelerated cell cycle progression so that much fewer cells could be arrested at M phase, thus precluding us from obtaining cells that are arrested at M with a lower mitochondrial mass. This result is consistent with previous reports showing that tightly regulated mitochondrial function and redox signaling are required for proper cell cycle progression and cell division [55]. Mitochondrial biogenesis and function, on the other hand, are also subjected to regulation by cell cycle regulators. Cdk1 was reported to promote protein import into mitochondria- in yeast, as a preparation for subsequent segregation into daughter cells [56]. Cyclin B1/CDK1 functions to phosphorylate mitochondrial proteins and augment ATP generation to promote G2 to M transition and shorten cell cycle time in mammalian cells [55]. Consistently, inhibition of CDK1 by RO-3306 led to G2 arrest [57]. These results indicate that mitochondrial dynamics/function and cell cycle progression are tightly coordinated. It is interesting to note that in HeLa cells, while the total cell volume may change during cell cycle, the mitochondria constantly occupy ~10–11% of the cytoplasmic volume by adjusting their own growth and division [58]. Forced reduction of mitochondrial content, as in the cases of CCCP treatment and PGC-1α depletion, may have compromised the mitochondrial readiness for cell cycle progression and result in fewer cells reaching and being arrested at M phase and thus alleviate mitochondrial stress associated with M arrest (Fig. 8H). Myc and mTOR act as key drivers of cell growth and biosynthesis. Myc inactivation can induce diapause-like state in cancer cells and confer them resistance to chemotherapy [59]. Colorectal cancer cells can also enter a diapause-like drug-tolerant persister state to evade death from chemotherapy [60]. We postulate that deceleration of cell cycle that confers resistance to chemotherapy may resembles the so-called diapause-like state. Cells trapped in a diapause-like state can presumably evade M-arrest and the consequent mitochondrial stress.

## Materials and methods

### Cell culture and treatment

HT1080 (fibrosarcoma, p53 wild-type), U2OS (osteosarcoma, p53 wild-type) and MCF-7 (breast cancer, p53 wild-type) cell lines were obtained from the Cell Bank of Chinese Academy of Sciences (Shanghai). All cell lines were authenticated using short tandem repeat profiling. TH287 (Selleck Chemicals, Houston, TX, USA) and UCN-01 (Sigma-Aldrich, St. Louis, MO, USA). The final concentration of DMSO in the culture medium was less than 0.05% (v/v). Control cultures received the same amount of DMSO. N-acetylcysteine (NAC) was purchased from Beyotime (China) and dissolved in water to make stock solutions (0.5 M, pH = 7.4). Mitoquinone (MitoQ) was purchased from MCE (USA) and dissolved in water to make stock solution (1 mM). Paclitaxel, also from MCE, was dissolved in DMSO for stock solution (50 μM). Carbonyl cyanide 3-chlorophenylhydrazone (CCCP) was purchased from MultiSciences Biotech (China) and dissolved in DMSO to make stock solutions (50 mM). Minimum Essential Medium (MEM), Dulbecco’s Modified Eagle Medium (DMEM) and fetal bovine serum were purchased from Gibco. The cells were maintained in DMEM or MEM supplemented with 10% FBS (Gibco,Thermo Fisher Scientific, Waltham, MA, USA), 100 U/mL penicillin, and 100 μg/mL streptomycin in a humidified 5% CO_2_/95% air atmosphere at 37 °C.

### Cell cycle analysis

For immunofluorescence analysis, cells grown on coverslips were fixed in Immunol Staining Fix Solution (Beyotime) for 15 min, washed with PBS once permeabilized with 0.25% Triton X-100 in PBS, and then blocked in Immunol Staining Blocking Buffer (Beyotime) for 1 h at room temperature, incubated with 1 μg/mL anti-pH3 antibody (06-570, Millipore) for 2 h at 4 °C, followed by a secondary antibody conjugated to AlexaFluor 488 (diluted 1: 200; Jackson ImmunoResearch Laboratories, West Grove, PA) for 1 h at 4 °C. Cells were washed in PBS and counterstained with 4,6-diamidino-2-phenylindole (DAPI)(Abcam,Cambridge, MA, USA). The coverslips were mounted on slides for examination. Images were taken using an Olympus DP71 fluorescence microscope.

For flow cytometer analysis, control and treated cells were harvested using 0.25% Trypsin–EDTA, centrifuged (400 × g) for 5 min, and washed once with cold PBS. The cells were fixed in 5 mL of cold 70% ethanol at −20 °C overnight. The fixed cells were washed with PBS once, permeabilized with 0.25% Triton X-100 in PBS, and then blocked in Immunol Staining Blocking Buffer for 1 h at room temperature, incubated with 1 μg/mL anti-pH3 antibody (06-570, Millipore) for 2 h at 4 °C, followed by a secondary antibody conjugated to AlexaFluor 488 (diluted 1:200; Jackson ImmunoResearch Laboratories, West Grove, PA) for 1 h at 4 °C. DNA was stained with 10 μg/mL propidium iodide (Beyotime) in the presence of 100 μg/mL RNaseA (Invitrogen) at room temperature for 30 min. Cell cycle distribution was analyzed by measuring DNA content using a BD Biosciences FACScan II cytometer (Becton Dickinson, San Jose, CA, USA). At least 10,000 cells were collected. The mitotic index is represented by the ratio of p-H3 positive cells/total number of cells. The data presented are the mean ± standard deviation (S.D.).

### Western blotting analysis

Equal amounts (30–50 μg) of proteins were separated by 12% SDS-PAGE, transferred to PVDF membrane (Millipore), and blocked with 5% nonfat dry milk in TBS-Tween 20 (0.1%, v/v) for at least 1 h at room temperature. The membrane was incubated with specific primary antibodies at 4 °C for overnight. After washing, the membrane was incubated with the appropriate horseradish peroxidase secondary antibody (diluted 1: 5000; Jackson ImmunoResearch Laboratories) for 1 h. Following three times washes, the blots were developed by ECL kit (Thermo). The antibodies used for Western blotting were anti-p-CHK1-Ser345 (ab58567, abcam, 1:1000), anti-CHK1 (sc-8408, Santa Cruz, 1:200), anti-γ-H2AX (#9718, CST, 1:1000), anti-OXPHOS (ab110411, abcam, 1:1000), anti-COX-IV (11242-1-AP, proteintech, 1:1000), anti-Acetylated-Lysine (#9441, CST, 1:1000), anti-SUMO1 (ab32058, abcam, 1:1000), anti-Cytochrome C (ab13575, abcam, 1:1000), anti-Cytochrome C (#12963, CST, 1:1000), anti-PGC-1α (66369-1-Ig, proteintech, 1:1000), anti-p-AKT-Ser473 (#4060, CST, 1:1000), anti-AKT (#9272, CST, 1:1000), anti-p-ERK (#4370, CST, 1:1000), anti-ERK (#4695, CST, 1:1000), anti-CyclinD (26939-1-AP, proteintech, 1:1000), anti-p21(ab109520, abcam, 1:1000), anti-GAPDH (10494-1-AP, proteintech, 1:10000), anti-α-Tubulin (66031-1-lg, proteintech, 1:10000), The protein levels were normalized by GAPDH or α-Tubulin.

### RNA interference

CHK1 and PGC-1α transient silencing was performed with siRNAs purchased from Sigma-Aldrich; CHK1-1#: sense 5′-CGGUCCCUGUACUCAGAAATT-3′, CHK1-2#: sense 5′-GAAGCAGUCGCAGUGAAGA-3′; PGC-1α-1#: sense 5′-CCUGUUUGAUGACAGCGAATT-3′, PGC-1α-2#: sense 5′-GGACAGUGAUUUCAGUAAUTT-3′; For control experiments, cells were transfected with a similar amount of non-targeting control siRNA (Sigma-Aldrich). Transient transfections of siRNAs were performed using lipofectamine 2000 (Invitrogen) according to the manufacturer’s instruction.

### Measurements of mitochondrial and cellular ROS

Mitochondrial ROS production was measured using Mito-SOX™ Red mitochondrial superoxide indicator (Invitrogen). Cells were seeded on coverslips and washed with PBS, and then pre-incubated with 1 μM Mito-SOX Red in PBS for 20 min at 37 °C in the dark. After treatments, Hoechst 33342 (KeyGEN BioTECH, China) was added for 20 min. The coverslips were mounted on slides for examination. Images were taken using an Olympus DP71 fluorescence microscope.

For flow cytometer analysis of intracellular ROS levels, cells were washed and harvested in PBS, and then separately stained with 1 μM Mito-SOX Red, 5 μM dihydroethidium (DHE), 10 μM DCFH-DA for 20 min at 37 °C and 5% CO_2_ in the dark. Samples were subsequently washed using ice-cold PBS and centrifuged for 5 min at 1500 rpm before being resuspended in ice-cold PBS and kept on ice until analysis. Flow cytometry was performed using a BD Biosciences FACScan II cytometer (Becton Dickinson, San Jose, CA, USA). At least 10,000 cells were collected.

### Comet assay

Comet assay was performed as previously described [61]. Cells were treated with TH287(10 μM) alone or in combination with UCN-01(300 nM) for 24 h. A CometAssay™ reagent kit for single-cell gel electrophoresis (Trevigen, Gaithersburg, MD, USA) was used to detect DNA damage according to the manufacturer’s protocol. Briefly, cells were harvested and mixed with low-melting LMAgarose. Immerse slides in 4 °C Lysis Solution for overnight. Electrophoresis was performed at 21 V for 40 min. Slides were stained with DAPI for 10 min. One hundred randomly selected cells per sample were captured under an Olympus DP71 fluorescence microscope. In this assay, the relative length and intensity of DNA tails relative to heads is proportional to the amount of DNA damage in individual nuclei. These parameters were measured by Olive tail moment with TriTek Comet Score software (TriTek Corp., Sumerduck, VA, USA).

### Apoptosis analysis

Apoptotic cells were identified using Annexin V/Dead Cell Apoptosis Kit (Invitrogen). Briefly, both adherent and floating cells were harvested, washed twice with ice-cold PBS. Then cells were resuspended in 100 μL of 1× annexin-binding buffer and incubated at room temperature for 15 min in the dark with annexin-V- Phycoerythrin and propidium iodide. Flow cytometry was performed using a BD Biosciences FACScan II cytometer (Becton Dickinson, San Jose, CA, USA). At least 10,000 cells were collected.

### Clonogenic assay

Single-cell suspensions were generated for HT1080 cells and 500 cells per plate were seeded into six-well tissue culture plates. Cells were treated as indicated, and then the medium was aspirated and fresh medium was added. After 2 weeks, colonies were stained with crystal violet. Colonies of greater than 50 cells were counted to determine the surviving fraction.

### Transmission electron microscopy

Transmission electron microscopy was performed as described previously [62]. HT1080 cells were washed with 0.1 M phosphate buffer (PB) and fixed with 1% OsO_4_ (osmium tetroxide) in PB for 2 h. After washing in 0.1 M PB, the fixed cells were dehydrated in a graded alcohol series and embedded in Epon epoxy resin. Ultrathin sections (60 nm) were prepared with diamond knives on a Leica EM UC7 microtome. Ultrathin sections were stained with uranyl acetate and lead citrate, and examined under an electron microscope (HT7700, Hitachi, Japan). Quantification of mitochondria was performed as follows. Number of mitochondria per field was counted from electron micrographs in each group.

### Measurement of mitochondrial mass

The cell-permeant MitoTracker™ Red CM-H2Xros probes (Invitrogen) contain a mildly thiol-reactive chloromethyl moiety for labeling mitochondria. For immunofluorescence analysis, cells were seeded on coverslips and washed with PBS, and then stained with 100 nM MitoTracker™ Red CM-H2Xros probe for 20 min at 37 °C and 5% CO_2_ in the dark. After treatments, Hoechst 33342 was added for 20 min. The coverslips were mounted on slides for examination. Images were taken using an Olympus DP71 fluorescence microscope.

For flow cytometer analysis, cells were washed and harvested in PBS, and then separately stained with 100 nM MitoTracker™ Red CM-H2Xros probe, 2.5 μM NAO(GENMED) for 20 min at 37 °C and 5% CO_2_ in the dark. Samples were subsequently washed using ice-cold PBS and centrifuged for 5 min at 1500 rpm before being resuspended in ice-cold PBS and kept on ice until analysis. Flow cytometry was performed using a BD Biosciences FACScan II cytometer (Becton Dickinson, San Jose, CA, USA). At least 10,000 cells were collected.

### Mitochondrial extraction

Mitochondria and cytosolic fractions were isolated with the Cell Mitochondria Isolation Kit (Beyotime) according to the manufacturer’s instructions. HT1080 cells were washed and harvested in ice-cold PBS, and mitochondria were extracted in a homogenizer in mitochondrial lysis buffer, followed by centrifugation at 1000 × g for 10 min at 4 °C. The supernatant was further centrifuged at 11,000 × g for 15 min at 4 °C to pellet the mitochondria. The pellets were collected as the mitochondrial fraction.

### Determination of mitochondrial DNA copy number

Total DNA was isolated from the cells using a FastPure Cell/Tissue DNA Isolation Mini Kit (Vazyme, China) according to the manufacturer’s instructions. To evaluate the mtDNA content, the relative amounts of mtDNA-coded Cytochrome Oxidase I (Forward: 5′-TCGCCATCATATTCGTAGGAG-3′, Reverse: 5′-GTAGCGTCGTGGTATTCCTGA-3′) were determined by the △△Ct method using nuclear DNA-coded β2-microglobulin (Forward: 5′-TTAACGTCCTTGGCTGGGTC-3′, Reverse: 5′-ACTGGAAGACAAAGGGCTCG-3′) as an internal control.

### Measurement of mitochondrial transmembrane potential (ΔΨm)

Mitochondrial depolarization was monitored with the potentiometric dye JC-1 using the Mitoprobe JC-1 assay kit (Thermo Fisher Scientific) according to the manufacturer’s instructions. JC-1 is a cationic, positively charged fluorescent dye that exhibits potential-dependent accumulation in mitochondria, indicated by a fluorescence emission shift from green (~525 nm) to red (~590 nm). Consequently, the mitochondrial depolarization is indicated by a decrease in the red/green fluorescence intensity ratio. HT1080 cells were stained with JC-1 (2 μM) for 30 min at 37 °C in 5% CO_2_, washed, resuspended in ice-cold PBS and kept on ice until analysis and then red/green fluorescence was monitored using a BD Biosciences FACScan II cytometer (Becton Dickinson, San Jose, CA, USA). At least 10,000 cells were collected.

### Measurement of oxygen consumption rate and glycolytic capacity

Seahorse metabolic analysis was carried out as described previously [63]. The cellular OCR and ECAR were determined using the Seahorse XFe 96 Extracellular Flux Analyzer (Seahorse Bioscience). The cells were evenly seeded in XF 96 cell culture microplate and allowed to attach for 24 h. OCR was measured in XF medium containing 1 mM sodium pyruvate, 2 mM glutamine and 10 mM glucose in basal conditions and ECAR was measured in XF medium containing 1 mM glutamine in basal conditions. Cells were washed with assay medium and then incubated at 37 °C in a non-CO_2_ incubator for 60 min. After baseline measurements, for OCR, oligomycin (ATP-synthase-inhibitor, 1 μM), FCCP (mitochondrial uncoupler, 4 μM), Rotenone (mitochondrial complex I inhibitor), and antimycin A (mitochondrial complex III inhibitor)(1 μM) were sequentially injected. Indices of mitochondrial function were calculated as Basal OCR (baseline OCR - Rotenone and antimycin A OCR), ATP-linked OCR(basal respiration rate - oligomycin OCR), Maximal respiration (FCCP OCR - Rotenone and antimycin A OCR). Glucose (10 mM), oligomycin (1 μM), and 2-DG (glycolytic inhibitor,100 mM) were sequentially injected into each well at the indicated time points, for the measurements of ECAR associated with glycolysis, glycolytic capacity and glycolytic reserve. Experiments with the Seahorse system have been performed with the following assay conditions: 3-min mixture; 3-min wait; and 3-min measurement; metabolic parameters were then calculated. Data are expressed as mean ± standard deviation (S.D.).

### Tumor xenografts in nude mice

Four- to six-week-old male nude mice were purchased from Beijing Experimental Animal Center and kept in pathogen-free conditions and handled in accordance with the requirements of the Guideline for Animal Experiments. The animals were subcutaneously inoculated with 2.5 × 10^6^ HT1080 cells (suspended in 100 µL PBS). Animals were randomly divided into four groups (*n* = 6 for each group). TH287 was administered at a fixed dose of 6 mg/kg and UCN-01 as 3 mg/kg and formulations were administered three times with a span of 2 days. The tumor size was measured everyday once until 16th day. Tumor growth was monitored with a caliper, and tumor volume was calculated according to the formula *V* = 1/2 maximal diameter × perpendicular diameter^2^. All nude mouse experiments were approved by the Institutional Animal Care and Use Committee of Shandong University.

### Immunohistochemistry

Mouse tissues were fixed in 4% paraformaldehyde (Servicebio, China) for 24 h, dehydrated, and embedded in paraffin blocks. Paraffin sections (4 μm) were deparaffinized in dimethylbenzene, and hydrated in a series of graded alcohol dilutions. Paraffin sections were immersed in EDTA Antigen Retrieval solution (ZSGB-Bio, China) and boiled in a microwave at 95–100 °C for 20 min, and subsequently cooled at room temperature for 30 min. Endogenous peroxidase was blocked with 3% H_2_O_2_ in PBS, and then blocked in 10% normal goat serum at 37 °C for 60 min. Sections were incubated with primary antibodies anti-phospho-H2AX (20E3, CST, 1: 100), anti-NDUFB8 (ab192878, abcam, 1:500), anti-Cytochrome C (ab13575, abcam, 1:1000) overnight at 4 °C. After washing, Sections were incubated with horseradish peroxidase conjugated secondary antibodies (ZSGB-Bio) for 30 min. The area of the immunocomplex was stained by chromogen 3, 3′- diaminobenzidine for 5 min. All the sections were lightly counterstained with hematoxylin before mounting. For the quantification of immunohistochemistry staining, randomly selected stained slides from six mice in each group and at least six photos were quantified by ImageJ software for each treatment arm. The positive staining in each slide was scored and presented as the relative expression level (the protein expression level in Control was arbitrarily set to base level of 1). Data were expressed as mean ± S.D.

### EdU incorporation assay

EdU incorporation assays were performed using Cell-Light EdU Cell Proliferation Detection kit (RiboBio) according to the manufacturer’s instructions. HT1080 cells were seeded on coverslips and were incubated with 50 μM EdU for 2 h. The cells were fixed in PBS containing 4% paraformaldehyde for 30 min, and then exposed to 2 mg/mL glycine for 5 min. After washing with PBS, the cells were incubated with 1 × Apollo staining solution for 30 min. The staining solution was discarded, and the cells were washed with PBS containing 0.5% Triton X-100 for 10 min. Cells were washed in PBS and counterstained with DAPI. The coverslips were mounted on slides for examination. Images were taken using an Olympus DP71 fluorescence microscope.

### Statistical analysis

All statistical data are presented as mean ± S.D. ANOVA was used to compare significant differences among multiple experimental groups. Two-sided Student’s *t* test was used for comparisons between two groups of experiments. *p* < 0.05 was considered statistically significant. Statistical analyses were carried out using GraphPad 7.00 software (GraphPad Software, La Jolla, CA, USA). * indicates *p* < 0.05, and ** indicates *p* < 0.01, *** indicates *p* < 0.001, **** indicates *p* < 0.0001, ns stand for no significant.

## Supplementary information

Legends to Supplementary Figures

Supplementary Figure 1

Supplementary Figure 2

Supplementary Figure 3

Supplementary Figure 4

Supplementary Figure 5

Supplementary Figure 6
