# Supplementary material for: Disrupted mitochondrial homeostasis coupled with mitotic arrest generates antineoplastic oxidative stress
Source: Oncogene. 2021 Nov 12;41(3):427–43. doi: 10.1038/s41388-021-02105-9 (PMC8755538; doi:10.1038/s41388-021-02105-9)
Supplement: Supplementary file 1 — Legends to Supplementary Figures [file 41388_2021_2105_MOESM1_ESM.doc]

**Supplementary Figure Legends**

**Supplementary Figure 1. TH287 induces M arrest and mitochondrial oxidative stress.**  (A) U2OS cells or (B) MCF-7 cells were treated with DMSO or TH287 (10 μM) for 24 h and then subjected to cell cycle distribution analysis by flow cytometry. Difference in proportions of G2/M-phase cells was statistically analyzed. (C) Left, representative immunofluorescence imaging of phosphorylated Histone H3(pH3) in U2OS cells treated with DMSO or TH287 (10 μM) for 24 h. Scale bar, 5 μm. Right, representative profiles of U2OS cells treated with DMSO or TH287 (10 μM) for 24 h assessed by pH3/PI FACS. The mitotic index (percentage of pH3-positve cells) for each group was shown. (D) MCF-7 cells were treated with DMSO or TH287 (10 μM) for 24 h and subjected to dual pH3/PI FACS. The mitotic indices were statistically analyzed. (E) Flow cytometric analysis of intracellular ROS levels measured by DCFH-DA. U2OS cells were treated with DMSO or TH287(10 μM) for 24 h. (F) Flow cytometric analysis of superoxide measured by dihydroethidium (DHE). U2OS cells were treated with DMSO or TH287(10 μM) for 24 h. (G) U2OS cells were treated with DMSO or TH287(10 μM) for 24 h. Mitochondrial superoxide was measured using MitoSOX Red, detected by flow cytometric analysis. (H) U2OS cells were pretreated with DMSO or UCN-01 (300 nM) for 4 h before addition of TH287 (10 μM) treatment for 24 h, and subjected to cell cycle distribution analysis by flow cytometry. The proportions of G2/M-phase cells were statistically analyzed. (I) Left, representative immunofluorescence imaging of pH3 in U2OS cells pretreated with DMSO or UCN-01 (300 nM) for 4 h followed by treatment with TH287 (10 μM) for an additional 24 h. Scale bar, 5 μm. Right, representative cell-cycle profiles of U2OS cells pretreated with DMSO or UCN-01 (300 nM) for 4 h followed by treatment with TH287 (10 μM) for an additional 24 h assessed by pH3/PI FACS. The mitotic indices were statistically analyzed. (J) Flow cytometric analysis of intracellular ROS levels measured by DCFH-DA. U2OS cells were treated with TH287 (10 μM) alone or in combination with UCN-01 (300 nM) for 24 h. (K) Flow cytometric analysis of superoxide measured by dihydroethidium (DHE). U2OS cells were treated with TH287 (10 μM) alone or in combination with UCN-01 (300 nM) for 24 h. (L) U2OS cells were treated with TH287 (10 μM) alone or in combination with UCN-01 (300 nM) for 24 h. Mitochondrial superoxide was detected by flow cytometric analysis after staining with MitoSOX Red. (M) U2OS cells were treated with TH287 (10 μM) alone or in combination with UCN-01 (300 nM) for 24 h. Mitochondrial superoxide, indicated by MitoSOX Red, was detected using fluorescence microscope. Scale bar, 5 μm. The data shown are presentative of three independent experiments and data presented in bars show the mean ± S.D.. The statistical differences between two groups were analyzed by two-sided unpaired Student's t-test. * p< 0.05, ** p< 0.01, *** p< 0.001, **** p< 0.0001.

**Supplementary Figure 2. Paclitaxel induces M arrest and mitochondrial oxidative stress.** (A) HT1080 cells were transfected with non-targeted control siRNA or CHK1-directed siRNA for 48 h. Subsequently, cells were treated with 10 μM TH287 for 24 h , and then subjected to cell cycle distribution analysis by flow cytometry. The proportions of G2/M-phase cells were statistically analyzed. RNAi efficiency of CHK1 expression was shown on the top. (B) HT1080 cells were transfected with non-targeted control siRNA or CHK1-directed siRNA for 48 h. Subsequently, cells were treated with 10 μM TH287 for 24 h and mitotic index was assessed by pH3/PI FACS. The mitotic indices were statistically analyzed. (C) HT1080 cells were transfected with non-targeted control siRNA or CHK1-directed siRNA for 48 h. Subsequently, cells were treated with 10 μM TH287 for 24 h, and intracellular ROS levels were then measured by flow cytometry. (D) HT1080 cells were treated as in (C), mitochondrial superoxide levels were then measured by flow cytometry. (E) HT1080 cells were pretreated with DMSO or UCN-01 (300 nM) for 4 h before paclitaxel (50 nM) treatment for 24 h and were then subjected to cell cycle distribution analysis by flow cytometry. The proportions of G2/M-phase cells were statistically analyzed. (F) Left, Representative immunofluorescence imaging of pH3 in HT1080 cells pretreated with DMSO or UCN-01 (300 nM) for 4 h followed by treatment with paclitaxel (50 nM) for an additional 24 h. Scale bar, 5 μm. Right, representative cell-cycle profiles of HT1080 cells pretreated with DMSO or UCN-01 (300 nM) for 4 h followed by treatment with paclitaxel (50 nM) treatment for an additional 24 h assessed by pH3/PI FACS. The mitotic indices were statistically analyzed. (G) Flow cytometric analysis of intracellular ROS levels measured by DCFH-DA. HT1080 cells were treated with paclitaxel (50 nM) alone or in combination with UCN-01 (300 nM) for 24 h. (H) HT1080 cells were treated with paclitaxel (50 nM) alone or in combination with UCN-01 (300 nM) for 24 h. Mitochondrial superoxide was detected by flow cytometric analysis after staining with MitoSOX Red. (I) and (J) HT1080 and U2OS cells were treated with paclitaxel (50 nM) alone or in combination with UCN-01 (300 nM) for 24 h. Mitochondrial superoxide, indicated by MitoSOX Red, was detected using fluorescence microscope. Scale bar, 5 μm. The data shown are representative of three independent experiments and data presented in bars indicate mean ± S.D.. The statistical differences between the two groups were analyzed by two-sided unpaired Student's t-test. ** p< 0.01, *** p< 0.001, **** p< 0.0001.

**Supplementary Figure 3. Depletion of CHK1 abolishes M arrest-coupled mitochondrial accumulation induced by TH287 or paclitaxel.** (A) HT1080 cells were transfected with non-targeted control siRNA or CHK1-directed siRNA for 48 h. Subsequently, cells were treated with 10 μM TH287 for 24 h and were stained with MitoTracker Red and analyzed using a flow cytometer. (B) U2OS cells were transfected with non-targeted control siRNA or CHK1-directed siRNA for 48 h. Subsequently, cells were treated with 10 μM TH287 for 24 h and were stained with MitoTracker Red and analyzed using a flow cytometer. RNAi efficiency of CHK1 expression was shown on the left. (C) HT1080 cells were transfected with non-targeted control siRNA or CHK1-directed siRNA for 48 h. Subsequently, cells were treated with 50 nM paclitaxel for 24 h and were stained with MitoTracker Red and analyzed using a flow cytometer. (D) HT1080 cells were transfected with non-targeted control siRNA or CHK1-directed siRNA for 48 h. Subsequently, cells were treated with 10 μM TH287 for 24 h. Mitochondria fractions were isolated with the Cell Mitochondria Isolation Kit. The intact mitochondria were isolated from 20 million treated cells and mitochondrial proteins were quantified using BCA Protein Assay. Equal amounts of proteins were loaded in each lane. The mitochondrial nature of the isolated proteins was confirmed with Western blotting analysis using antibodies against various mitochondrial components. (E) HT1080 cells were transfected with non-targeted control siRNA or CHK1-directed siRNA for 48 h. Subsequently, cells were treated with 50 nM paclitaxel for 24 h. Western blotting analysis using antibodies against various mitochondrial components were treated as described in (D). (F) Mitochondrial DNA (mtDNA) copy number measured by quantitative PCR. The relative amounts of Cytochrome Oxidase I (COX I) in total DNA were determined as described in Materials and methods. The data shown are representative of three independent experiments and data presented in bars as mean ± S.D.. The statistical differences between the two groups were analyzed by two-sided unpaired Student's t-test. *** p< 0.001, **** p< 0.0001.

**Supplementary Figure 4. TH287 and paclitaxel increase SUMOylation and acetylation of mitochondrial proteins.** (A) Mitochondria were isolated from HT1080 cells treated with TH287 (10 μM) alone or TH287 in combination with UCN-01 (300 nM) for 24 h. Antibody to ATP5A were incubated with HT1080 cells mitochondrial extracts. Immunoprecipitates were resolved on SDS-PAGE and probed for SUMO1-conjugated proteins. (B) Mitochondria were isolated from HT1080 cells treated with paclitaxel (50 nM) alone or paclitaxel in combination with UCN-01 (300 nM) for 24 h. Antibody to ATP5A were incubated with HT1080 cells mitochondrial extracts. Immunoprecipitates were resolved on SDS-PAGE and probed for SUMO1-conjugated proteins. (C) Mitochondria were isolated from HT1080 cells treated with TH287 (10 μM) alone or TH287 in combination with UCN-01 (300 nM) for 24 h. Antibody to Cyto C were incubated with HT1080 cells mitochondrial extracts. Immunoprecipitates were resolved on SDS-PAGE and probed for Acetyl-conjugated proteins. (D) Mitochondria were isolated from HT1080 cells treated with paclitaxel (50 nM) alone or paclitaxel in combination with UCN-01 (300 nM) for 24 h. Antibody to Cyto C were incubated with HT1080 cells mitochondrial extracts. Immunoprecipitates were resolved on SDS-PAGE and probed for Acetyl-conjugated proteins. Data shown are representative of three independent experiments .

**Supplementary Figure 5. M arrest is coupled with increased mitochondrial biogenesis.** (A) Western blotting analysis of PGC-1α protein levels in HT1080 cells treated with paclitaxel (50 nM) alone or paclitaxel in combination with UCN-01 (300 nM) for 24 h. (B) HT1080 cells were transfected with non-targeted control siRNA or PGC-1α-directed siRNA for 48 h. Subsequently, cells were treated with 50 nM paclitaxel for 24 h , and then subjected to cell cycle distribution analysis by flow cytometry. The proportions of G2/M-phase cells were statistically analyzed. (C) HT1080 cells were transfected with non-targeted control siRNA or PGC-1α-directed siRNA for 48 h. Flow cytometric analysis of intracellular ROS levels measured by DCFH-DA in HT1080-siSCR and HT1080-siPGC-1α cells treated with paclitaxel (50 nM) for 24 h. (D) HT1080 cells were transfected with non-targeted control siRNA or PGC-1α-directed siRNA for 48 h. Flow cytometric analysis of Mitochondrial superoxide levels by MitoSOX Red in HT1080-siSCR and HT1080-siPGC-1α cells treated with paclitaxel (50 nM) for 24 h. (E) HT1080 cells were transfected with non-targeted control siRNA or siPGC-1α-directed siRNA for 48 h. Subsequently, cells were treated with 50 nM paclitaxel for 24 h and were stained with MitoTracker Red and analyzed using a flow cytometer. (F) HT1080 cells were transfected with non-targeted control siRNA or siPGC-1α-directed siRNA for 48 h. Subsequently, cells were treated with 50 nM paclitaxel for 24 h and Mitochondria fractions were isolated. The mitochondrial nature of the isolated proteins was confirmed with Western blotting analysis using antibodies against various mitochondrial components. Data shown are representative of three independent experiments and data presented in bars as mean ± S.D.. The statistical differences between the two groups were analyzed by two-sided unpaired Student's t-test. *** p< 0.001, **** p< 0.0001.

**Supplementary Figure 6. Abrogation of M arrest reduces genotoxicity of cancer drugs.** (A) Western blotting analysis of γ-H2AX levels in HT1080 cells treated with paclitaxel (50 nM) alone or in combination with UCN-01 (300 nM) for 24 h. (B) Western blotting analysis of γ-H2AX levels in HT1080 cells treated with paclitaxel (50 nM) alone or in combination with MitoQ (1 μM) for 24 h. (C) Clonogenic assay of HT1080 cells treated with paclitaxel (50 nM) alone or in combination with UCN-01 (300 nM). (D) Clonogenic assay of HT1080 cells treated with paclitaxel (50 nM) alone or in combination with MitoQ (1 μM). (E) Western blotting analysis of p-CHK1, total CHK1 and γ-H2AX protein levels in HT1080 cells pretreated with CCCP (12.5 μM) for 48 h before paclitaxel (50 nM) treatment for 24 h. Data shown are representative of three independent experiments and data presented in bars as mean ± S.D.. The statistical differences between the two groups were analyzed by two-sided unpaired Student's t-test. ** p< 0.01, *** p< 0.001, **** p< 0.0001.
